# An LC-MS/MS Method for Quantification of Lamotrigine and Its Main Metabolite in Dried Blood Spots

**DOI:** 10.3390/ph17040449

**Published:** 2024-03-30

**Authors:** Daniela Milosheska, Robert Roškar, Tomaž Vovk, Bogdan Lorber, Iztok Grabnar, Jurij Trontelj

**Affiliations:** 1 Faculty of Pharmacy, University of Ljubljana, Aškerčeva cesta 7, 1000 Ljubljana, Sloveniatomaz.vovk@ffa.uni-lj.si (T.V.);; 2 Department of Neurology, University Medical Centre Ljubljana, Zaloška cesta 2, 1000 Ljubljana, Slovenia

**Keywords:** dried blood spot, lamotrigine, lamotrigine glucuronide, therapeutic drug monitoring, clinical validation, haematocrit effect

## Abstract

Background: The antiepileptic drug lamotrigine (LTG) shows high pharmacokinetic variability due to genotype influence and concomitant use of glucuronidation inducers and inhibitors, both of which may be frequently taken by elderly patients. Our goal was to develop a reliable quantification method for lamotrigine and its main glucuronide metabolite lamotrigine-N2-glucuronide (LTG-N2-GLU) in dried blood spots (DBS) to enable routine therapeutic drug monitoring and to identify altered metabolic activity for early detection of drug interactions possibly leading to suboptimal drug response. Results: The analytical method was validated in terms of selectivity, accuracy, precision, matrix effects, haematocrit, blood spot volume influence, and stability. It was applied to a clinical study, and the DBS results were compared to the concentrations determined in plasma samples. A good correlation was established for both analytes in DBS and plasma samples, taking into account the haematocrit and blood cell-to-plasma partition coefficients. It was demonstrated that the method is suitable for the determination of the metabolite-to-parent ratio to reveal the metabolic status of individual patients. Conclusions: The clinical validation performed confirmed that the DBS technique is a reliable alternative for plasma lamotrigine and its glucuronide determination.

## 1. Introduction

**Figure 1:**
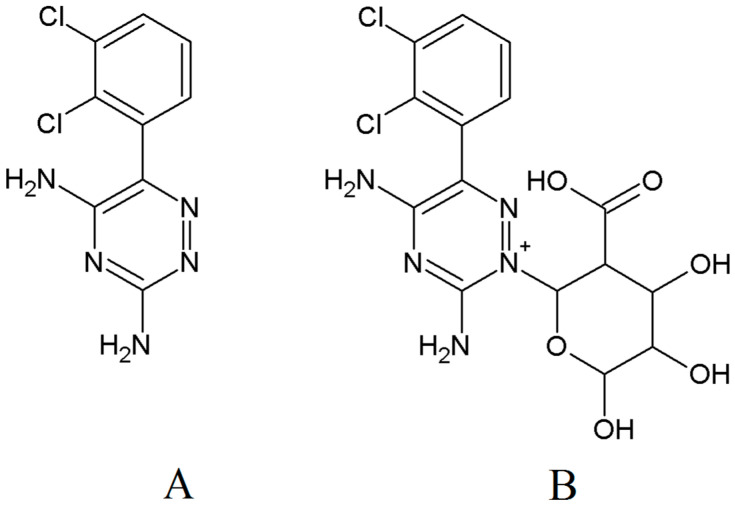
Chemical structures of (**A**) LTG and (**B**) LTG-N2-β-D-glucuronide.

Lamotrigine (LTG, 3,5-diamino-6-(2,3-dichlorophenyl)-1,2,4-triazine, Figure 1A) is a widely used second-generation antiepileptic drug approved for the treatment of focal and generalized seizures in adults and children [1,2]. Following oral administration, it is rapidly and completely absorbed. LTG undergoes extensive metabolism by glucuronidation, where 75% of the LTG dose is metabolized to the lamotrigine-N2-glucuronide (LTG-N2-GLU, Figure 1B), mainly by 1A4 and 2B7 isoforms of the uridine-diphosphate-glucuronosyltransferase (UGT) family. Excretion of the unconjugated LTG and its metabolites occurs via the urinary system [3].

LTG’s large variability in pharmacokinetics, the possibility of drug interactions and marked changes in drug concentrations due to conditions such as pregnancy, as well as the usage of concomitant UGT inducers (such as phenytoin, barbiturates, carbamazepine) and inhibitors (such as valproic acid), are some of the reasons for the need for therapeutic drug monitoring (TDM) [4,5]. As it was recently reported, hormone replacement therapy may significantly increase lamotrigine clearance due to the induction of UGT1A4 by estradiol, leading to the loss of therapeutic response [5,6]. There are many other possible UGT inhibitors and inducers taken by the elderly population, including over-the-counter medications and herbal supplements, as well as foods rich with seasoning like garlic (a UGT inducer) or turmeric and ginseng (UGT inhibitors) [7]. Therefore, it may be useful to routinely monitor the levels of lamotrigine. The quantification of the main metabolite is beneficial for recognizing the induction- or inhibition-mediated drug–drug interactions and genotypic variations. Dried blood spots (DBS) offer significant advantages over classic venepuncture in terms of reducing the number of hospital visits and patient burden and may therefore reduce healthcare costs [8]. The development of analytical methods employing the DBS sampling technique is quite challenging because of the smaller sample volumes and richer matrix due to the presence of whole blood. Validation of such methods requires special protocols to ensure adequate quality of the analytical results. Therefore, for the development of analytical methods for TDM by using DBS samples, it is necessary to overcome some major obstacles for their routine implementation [9]. 

Till present, several analytical methods have been reported for the analysis of LTG and/or its main metabolite in plasma or serum samples [10,11,12,13,14,15,16,17,18,19,20,21,22,23,24,25,26,27,28,29,30,31,32,33,34,35,36,37] as well as in dried blood [38,39,40,41,42,43,44,45,46,47,48]; however, there were no methods for the determination of lamotrigine together with its main metabolite in DBS. We consider the latter highly valuable for identifying the reasons for the changed LTG clearance in patients with sub- or supra-therapeutic plasma levels. The therapeutic range for LTG plasma concentrations is 2.5–15 µg/mL, while the range for its main metabolite has not yet been established. Monitoring the metabolite-to-parent ratio may reveal UGT induction or inhibition and allow differentiation from patient compliance issues [49]. 

Our objective was to develop and validate an LC-MS/MS method for the determination of LTG and its main metabolite, LTG-N2-GLU, in DBS samples according to the current guidelines and recommendations for DBS assays [40,41]. In this study, we present a successful validation and application of the developed method for the determination of whole blood concentrations of both analytes in patients with epilepsy on stable LTG therapy. To the best of our knowledge, this is the first clinically validated LC-MS/MS method for simultaneous analysis of LTG and its main metabolite in DBS samples in a range from 0.1 to 20 µg/mL that is suitable for routine clinical application. 

## 2. Results and Discussion

Our objective was to develop and validate a method for quantification of LTG and its main metabolite, LTG-N2-GLU, in DBS samples that combines the advantages of the DBS sampling technique and the LC-MS/MS method in terms of sensitivity, selectivity, and short analysis run time. Although several LC-MS/MS methods for lamotrigine quantitation in DBS samples exist that offer either a superior sensitivity [39] or include multiple other antiepileptic drugs [38,39,40,41,42,44,46,47,48], none of them offers the capability of simultaneous measurement of both LTG and LTG-N2GLU. During the optimization of sample preparation, preliminary experiments were performed with the HPLC-UV method that was used for the analysis of plasma samples, with some minor modifications. Different DBS card materials (Whatman^®^ 903, FTA DPMK-A, FTA DPMK, and FTA DPMK-C), extraction solvents (methanol, acetonitrile, methanol-water (90:10, *v*/*v*), acetonitrile-water (90:10, *v*/*v*), and 0.1% formic acid in methanol/water (90:10, *v*/*v*), volumes of extraction solvent (500, 750, 1000 µL), incubation times (15, 30, 60 min), and sonication times (10, 15, 20 min) were tested. Among various tested DBS card materials, Whatman^®^ 903 cards were selected due to the highest analyte recovery and lowest baseline signals. Despite optimization of several parameters of liquid extraction, many coelution peaks at the retention time of the glucuronide compromised its accurate and precise determination. To exclude the potential matrix effect on LC-MS/MS measurements, we decided to include an additional sample cleaning step with solid phase extraction. The cationic nature of analytes enabled the selection of strong mixed-mode sorbents (Strata-X-C), which employ both cationic and hydrophobic, as well as p−p interactions. The obtained samples revealed no interference with either the UV or MS/MS chromatographic methods.

### 2.1. Selectivity

**Figure 2:**
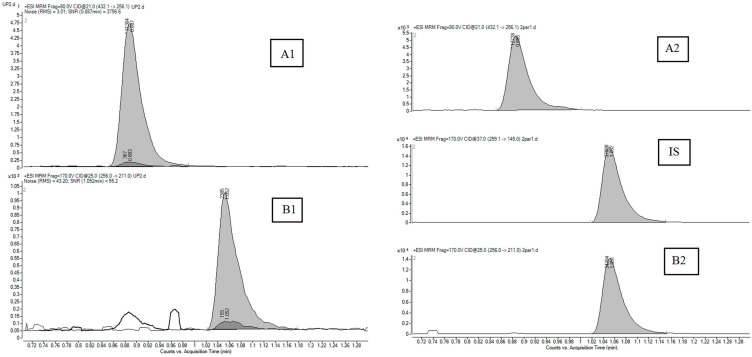
MS chromatograms recorded from a DBS sample at LLOQ (left-hand side) showing good signal-to-noise ratios for LTG-N2-GLU (**A1**) and LTG (**B1**). Overlaid chromatograms from a blank DBS sample show excellent selectivity without any matrix interferences ((**A1**,**B1**), dark grey areas). Typical DBS chromatograms obtained from a patient treated with LTG are presented on the right-hand side: LTG-N2-GLU (**A2**) and LTG (**B2**) with its internal standard (**IS**).

No interferences at the retention times of LTG and LTG-N2-GLU were detected. Representative blank chromatograms overlaid with DBS sample chromatograms at the concentration level of the LLOQ are shown in Figure 2. Typical DBS chromatograms obtained from a patient treated with LTG are presented on the right side.

### 2.2. Linearity

The linearity of the assay was confirmed on three consecutive days using the calibration standard solutions covering the concentration range from 0.1 to 20 µg/mL for both LTG and LTG-N2-GLU, showing a comparable sensitivity to that of the published plasma methods for simultaneous analysis of LTG and its metabolite [25,29]. The selected analytical range is wider than the proposed reference range for LTG (2.5–15 µg/mL), which confirms the suitability of the developed method for application in routine clinical practice for TDM as well as for pharmacological studies. The validated method was linear over the whole calibration range, with determination coefficients r^2^ > 0.993 obtained by non-weighted linear regression analysis. The LLOQ for both analytes was 0.1 µg/mL. The parameters of the calibration curves for LTG and LTG-N2-GLU and the corresponding regression coefficients are summarized in Table 1.

### 2.3. Accuracy and Precision

The intra- and inter-day accuracies of the method for LTG ranged from 3.7 to 10.6% and from −1.5 to 10.4%, respectively (Table 2). The intra- and inter-day accuracies for LTG-N2-GLU ranged from −6.0 to −0.2% and from −7.8 to 8.3%, respectively. All results agreed with the predefined acceptance criteria. The intra- and inter-day precisions of LTG and LTG-N2-GLU did not exceed 5.9 and 6.8%, respectively.

### 2.4. Haematocrit Effect and Influence of Blood Spot Volume

The haematocrit (Hct) is an important parameter that can influence DBS method performance. Recommendations are to identify the expected range of Hct values in the population of interest and validate this range by preparing batches at a minimum of two concentration levels. When the target Hct range results in analyte concentrations outside the predefined range, then a correction of the results based on the individual Hct values should be made [50]. In our case, based on calculated bias and CV values, no significant Hct effect on accuracy was observed either for LTG or its metabolite (Table 3).

Moreover, the analysis of the influence of blood volume showed no significant effect of the spot volume on the measured concentration for both analytes (CV less than 4.45% for LTG-N2-GLU and 4.90% for LTG) and confirmed the suitability of the assay performance for routine practice. 

### 2.5. Matrix Effect and Recovery

The overall mean recovery at low and high QC concentration levels was close to 100% and showed consistency and reproducibility, with CV values of less than 5.4% for LTG and 8.1% for LTG-N2-GLU. The comparison of slopes from five calibration curves prepared in different lots of matrices showed that the method is free from any significant relative matrix effects for LTG (RSD 3.0%) and for LTG-N2-GLU (RSD 1.8%). 

### 2.6. Stability

The results of the stability testing of the DBS cards presented as a percentage of a drug determined relative to those obtained from the freshly prepared samples are shown in Table 4. Autosampler stability testing showed that samples were stable at 5 °C for up to 24 h, with the mean bias stability less than 98.8 and 113.7% for LTG and LTG-N2-GLU, respectively. No significant changes from the reference concentration (t = 0) were observed during the stability testing period of DBS cards. The stability of stock solutions of LTG after 7 days of storage at 4 °C and 23 months of storage at −80 °C for LTG-N2-GLU was also confirmed. The results obtained from the stability testing of DBS cards were all within ±15% (Table 4).

### 2.7. DBS versus Plasma Concentrations and Clinical Application of the Method

Currently, the proposed reference ranges for AEDs are defined for plasma/serum samples. On the other hand, the measured concentrations in DBS samples represent drug concentrations in the whole blood. Since DBS and plasma concentrations can differ because of the drug partitioning between plasma and blood cells and measured drug concentrations in DBS samples can be significantly influenced by blood Hct, it is necessary to establish the relationship between the plasma and DBS concentrations for appropriate clinical interpretation of the results obtained from DBS samples [9,45]. The intention is to confirm that DBS samples are a valid alternative to conventional plasma samples for TDM and to determine the corresponding reference range for AEDs in DBS. 

**Figure 3:**
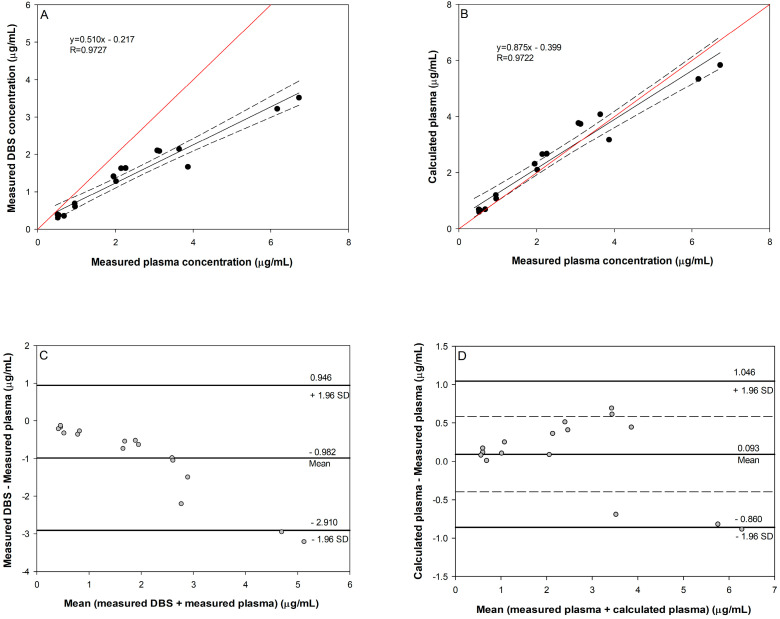
Comparison of LTG-N2-GLU DBS and blood plasma concentrations in nine patients. Weighted Deming regression of LTG-N2-GLU plasma concentrations plotted against the measured DBS concentration (**A**) or the calculated plasma concentrations using Equation (1) (**B**). The red line is the identity line. Bland–Altman plot for LTG-N2-GLU determined using the measured plasma and DBS (**C**) or the calculated concentrations using Equation (1). The mean line represents the bias between the measured DBS (**C**) or the calculated plasma (**D**) and measured plasma concentrations with a 95% confidence interval (dashed). The 1.96 SD lines indicate limits of the 95% agreement interval.

**Figure 4:**
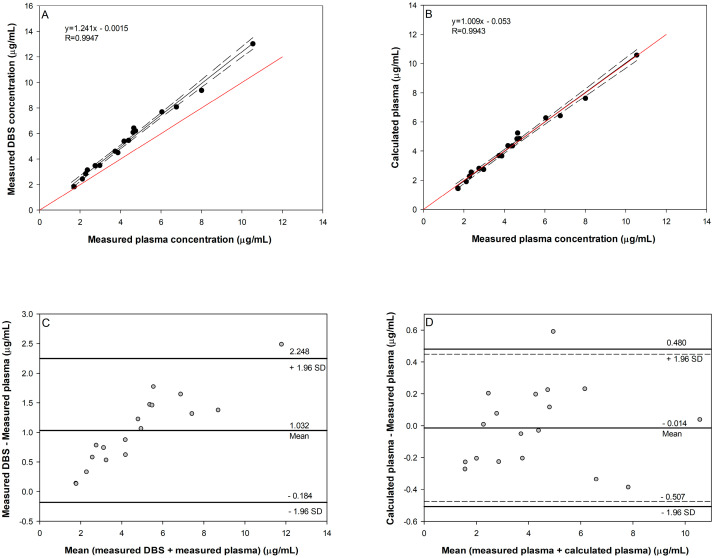
Comparison of LTG DBS and blood plasma concentrations in nine patients. Weighted Deming regression of LTG plasma concentrations plotted against measured DBS concentration (**A**) or calculated plasma concentrations using Equation (3) (**B**). The red line is the identity line. Bland–Altman plot for LTG determined using measured plasma and DBS (**C**) or calculated concentration using Equation (3). The mean line represents the bias between the measured DBS (**C**) or calculated plasma (**D**) and measured plasma concentrations with a 95% confidential interval (dashed). The 1.96 SD lines indicate limits of the 95% agreement interval.

In our study, clinical validation by comparison of the obtained results from DBS and plasma samples of the same patients obtained by venepuncture was performed. LTG and LTG-N2-GLU were quantified in 18 paired plasma and DBS samples from nine patients on stable LTG therapy. The measured concentrations in DBS samples were in the range of 1.83 to 12.95 µg/mL for LTG and from 0.31 to 3.52 µg/mL for LTG-N2-GLU, while the plasma concentrations ranged from 1.69 to 10.54 µg/mL and from 0.52 to 6.72 µg/mL for LTG and its metabolite, respectively. It is evident that the measured concentrations of LTG were higher in whole blood compared to plasma, which is in accordance with the previously published results [37,39,41]. The correlation between paired plasma and DBS concentrations was performed by weighted Deming regression, while a Bland–Altman plot was used to illustrate the agreement between the methods (Figure 3 and Figure 4).

The direct correlation between observed LTG-N2-GLU plasma and DBS concentrations, using a weighted Deming regression, is shown in Figure 3A. In the case of LTG-N2-GLU, the obtained slope was 0.510, while the intercept was 0.217. The slope of the Deming regression line presents the blood-to-plasma concentration ratio (R). For drugs with a low affinity for blood cells, R values are in the interval 0.4 to 0.6. It is recommended to consider the variation in Hct for such drugs in order to better correlate their DBS and plasma concentrations [10]. Plasma concentrations can be calculated using Equation (1), where C_P_ is plasma concentration, C_B_ is whole blood concentration, and Hct is haematocrit:

(1) $$c_{PL}=\frac{c_{B}}{1-Hct}$$

Parameters of the regression line for LTG-N2-GLU showed a good agreement between calculated (using Equation (1)) and observed plasma concentrations (Figure 3B). Moreover, the difference between calculated plasma concentrations using Equation (1) and the measured plasma concentration is small (0.093 µg/mL), and the 95% confidence interval of the mean difference (−0.397, 0.583) indicates that the difference between the two assessments is not significant at the 5% significance level (Figure 3D). These results confirmed the suitability of the method used for the calculation of LTG-N2-GLU plasma concentration by using whole blood concentrations and Hct.

On the other hand, the parameters of the weighted Deming regression line for LTG plasma and DBS concentrations were 1.241 and 0.0015 for the slope and intercept, respectively (Figure 4A). Here, it is evident that LTG has a higher affinity for blood cells than its metabolite, which is expected considering the more lipophilic nature of the LTG molecule. The concentration ratio between blood cells and plasma is determined by the blood cell-to-plasma partition coefficient (K_BC/PL_):

(2) $$K_{BC/PL}=\frac{c_{BC}}{c_{PL}}$$

K_BC/PL_ was estimated by nonlinear least squares fitting of Equation (3) to the measured values of Hct, C_B_, and C_PL_:

(3) $$c_{PL}=\frac{c_{B}}{(1-Hct)+K_{BC/PL}\times Hct}$$

The K_BC/PL_ value of 1.57 indicated that accounting only for Hct when calculating the LTG plasma concentration from the whole blood concentration is not satisfactory. The obtained slope value of 1.009 demonstrated a good agreement between the calculated (using Equation (3)) and measured plasma concentrations, which is shown in Figure 4B. Additionally, the Bland–Altman analysis of measured plasma and calculated plasma concentrations showed that the mean difference is very small (−0.014 µg/mL), and the 95% confidence interval of the mean difference (0.448, −0.476) indicated no significant difference between these two assessments (Figure 4C,D). The line of identity, with a slope of 1, lies within the 95% CI of the Deming regression line, which confirms that LTG DBS concentrations can be transformed into plasma concentrations by considering the values of Hct and K_BC/PL_. On the other hand, for the calculation of LTG-N2-GLU plasma concentration from DBS data, only Hct is needed since glucuronides have very limited cell membrane permeability and need transport proteins for distribution across cell membranes [51]; therefore, the K_BC/PL_ for LTG-N2-GLU can be classified as negligible, which is also supported by our results (Figure 3B).

Overall, we can conclude that the LTG and its metabolite plasma concentrations can be calculated from DBS concentrations with satisfactory precision by taking into account Hct and the partition between the plasma and blood cells. 

**Figure 5:**
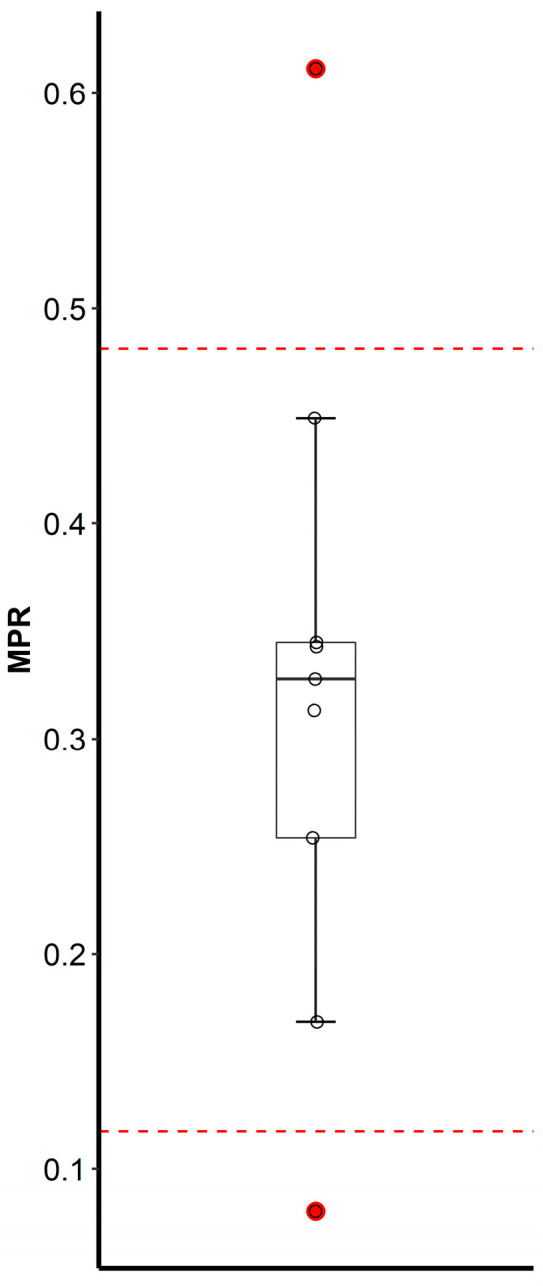
Boxplot of the metabolite-to-parent ratio (MPR) in trough DBS samples from a set of 9 patients on LTG therapy in steady-state with overlaid measurements in individual subjects (circles). Horizontal dashed lines are Tukey’s fences. Two outlying subjects are indicated by red-filled circles.

The simultaneous measurement of LTG-N2-GLU together with LTG offers a way to monitor the metabolite-to-parent ratio (MPR), which can be used to identify patients with altered metabolic clearance due to genotypic differences or induction/inhibition of the metabolism due to drug interactions. Unlike the measurement of the parent drug concentration alone, MPR can be used to differentiate patients with altered metabolic clearance from those who are poorly compliant with the prescribed treatment. In our study, we identified two patients (nr. 1 and nr. 4) with MPR outside Tukey’s fences (Grubbs test, *p* = 0.089), which indicates outlying data (Table 5 and Figure 5). One of these patients (nr. 4) was concomitantly taking phenytoin (a strong metabolic inducer). Indeed, the subject demonstrated a significantly elevated MPR of 0.611 compared to the mean of all subjects. Furthermore, subject nr. 4 also required a higher dose (400 mg/day) to achieve approximately the same through concentration of LTG, 3.5 µg/mL, as subjects taking 200–250 mg/day (subjects nr. 2, 3, and 8). Moreover, in subject nr. 4, the through concentration of LTG was almost three-fold lower compared to subject nr. 9, who took the same 400 mg/day dose, pointing to significantly induced metabolic clearance in subject nr. 4, likely due to the phenytoin-induced UGT enzyme activity [48]. Other antiepileptic drugs could also interfere with LTG pharmacokinetics, but they were not present in our study population. On the other side, the MPR of 0.080 in subject nr. 1 was considerably lower compared to the mean of all subjects. This could be related to inhibition of the metabolism by other concomitantly prescribed treatments or genetic variability resulting in decreased enzymatic activity. Our subsequent investigations revealed that patient nr. 1 was a carrier of the T allele of *UGT2B7*-161C>T (rs766825), which has been associated with decreased UGT2B7 activity [5]. 

To confirm the usefulness of the method for home sampling using the DBS approach, the venous plasma samples would need to be compared with the paired DBS samples obtained from capillary blood. Since capillary blood could not be obtained during the study, we worked with the venous blood. Regardless of this limitation, our method is applicable in hospitals where analytical methods for the quantification of LTG and LTG-N2-GLU are not available. In such cases, DBS samples could be prepared in the hospital from routinely collected venous blood and sent to external clinical laboratories. Such samples are stable at room temperature, so transportation via a cold chain is not required, as would be the case for venous plasma samples, which could reduce the analytical costs.

## 3. Materials and Methods

### 3.1. Materials

LTG was purchased from Sequoia Research (Pangbourne, UK), while its metabolite LTG-N2-GLU was obtained from Iris Biotech GmbH (Marktredwitz, Germany). The stable isotope labelled ^13^C_3_ lamotrigine used as an internal standard (IS) was obtained from Santa Cruz Biotechnology (Santa Cruz, CA, USA). All standards were of analytical grade. Suprapur^®^ formic acid and methanol for chromatography, Lichrosolv^®^ (MeOH), were obtained from Merck (Darmstadt, Germany) and Sigma Aldrich (Steinheim, Germany), respectively. All other chemicals: potassium dihydrogen phosphate, acetonitrile, formic acid, 25% ammonia solution, and 85% ortophosphoric acid, all from Merck (Darmstadt, Germany), were at least of analytical grade. Ultrapure water was obtained by A10 Advantage Milli-Q water purification system (Millipore Corp., Billerica, MA, USA). Six-millilitre blood collection tubes with EDTA were obtained from BD (New York, NY, USA). Solid phase extraction (SPE) cartridges Strata-X-C, 33 μm, 60 mg/3 mL were obtained from Phenomenex (Torrance, CA, USA). Whatman^®^ 903 blood spot cards were purchased from GE Healthcare (Piscataway, NJ, USA) and Minipax^®^ absorbent packets were purchased from Sigma-Aldrich (Steinhein, Germany).

### 3.2. Calibration Standards

The stock solutions of LTG, LTG-N2-GLU, and IS were prepared in methanol to yield concentrations of 1 mg/mL. A working standard solution containing LTG and LTG-N2-GLU at a concentration of 0.5 mg/mL was made by dilution with methanol. All solutions were stored in a freezer at −20 °C. An amount of 480 µL of drug-free venous blood from healthy donors collected into EDTA vacutainers was spiked with 20 µL of a combined standard solution of both LTG and LTG-N2-GLU prepared by appropriate dilutions with a solvent containing methanol-water (50:50, *v*/*v*) from the working solution. Spiked samples were left to equilibrate for 30 min at room temperature with occasional gentle shaking, and subsequently, 10 µL was spotted on the DBS card. Samples were dried at room temperature for at least 3 h and stored at room temperature in a sealed bag containing dry desiccant until analysis. DBS samples for calibration curve construction ranged from 0.1 to 20 µg/mL for both analytes at 11 calibration points (0.1, 0.25, 0.5, 0.75, 1.0, 5.0, 7.5, 10.0, 12.5, 17.5, 20.0). The quality control (QC) samples at low, medium, and high concentrations were prepared in a similar manner to yield 0.3, 3.0, and 15.0 µg/mL, respectively. DBS samples were further prepared as described in the chapter “DBS samples processing”. DBS calibration standards and QC standards were prepared daily and analysed the following day.

### 3.3. Patient Samples

Eighteen paired plasma and DBS samples were obtained from 9 patients with epilepsy on stable LTG therapy at the Department of Neurology, University Medical Centre Ljubljana, Slovenia, during their regular ambulatory visits. All patients enrolled in the study have signed a written informed consent. Adult patients with confirmed epilepsy on oral LTG treatment, either as a mono- or combination therapy, were eligible for the study. Chronic renal and hepatic diseases, as well as pregnancy, were exclusion criteria for the study. The study was carried out in accordance with the Declaration of Helsinki and was approved by the Slovenian National Medical Ethics Committee (grant 25p/04/12). Venous blood samples were collected in EDTA vacutainers immediately before the morning dose (trough concentration) and 2 to 4 h after dosing (peak concentration). Ten microlitre aliquots of the whole blood were precisely spotted onto the Whatman^®^ 903 cards and dried at room temperature for at least 3 h. DBS samples were stored at room temperature in a sealed bag containing dry desiccant. Samples were analysed within 30 days. The rest of the collected blood samples were afterwards centrifuged at 3000× *g* for 10 min, separated from the pellet, and stored at −80 °C until analysis.

### 3.4. Analysis of Plasma Samples

For plasma concentration measurements of LTG and LTG-N2-GLU, an adapted HPLC method with UV detection from Saracino et al. was used [27]. Briefly, a plasma aliquot of 165 µL was precipitated with 600 µL of ice-cold methanol, centrifuged, and the supernatant was evaporated to dryness. The dry residue was then reconstituted with 100 µL of mobile phase. The separation was performed on a C8 column (Zorbax^®^ Eclipse XDB-C8, 4.6 × 150 mm, 5 µm, Agilent Technologies, Santa Clara, CA, USA) using isocratic elution with potassium phosphate buffer (25 mM; pH 2.5)—methanol (80:20, *v*/*v*) as a mobile phase. The UV detector was set to 220 nm. The method was validated according to the FDA guidance for bioanalytical method validation [52]. The assay was calibrated over the concentration range of 0.1–20 µg/mL (r^2^ > 0.998) for LTG and 0.25–20 µg/mL (r^2^ > 0.997) for LTG-N2-GLU. The intra-day accuracies expressed as bias were from 1.0 to 4.0% and from 4.0 to 12.0%, for LTG and LTG-N2-GLU, respectively. The inter-day accuracies were from 4.0 to 7.0% for both analytes. The intra- and inter-day precisions were below 13.0% and 8.0% RSD, for LTG and LTG-N2-GLU, respectively.

### 3.5. DBS Samples Processing

The whole DBS spot (10 µL) was cut out with scissors from the DBS card into a 2 mL polypropylene tube. The sample was extracted with 500 µL methanol containing IS (0.2 µg/mL) at room temperature for 30 minutes. After the addition of 1.5 mL of 4% phosphoric acid solution, the sample was vortexed, sonicated for 15 min, and centrifuged at room temperature for 10 min at 16,000× *g*. An amount of 1.8 mL of the supernatant was loaded on Strata-X-C SPE cartridge which was previously conditioned with 2 mL of methanol and 2 mL of purified water. The loaded cartridge was washed with 1 mL of 2% formic acid and 1 mL of methanol and finally, the analytes were eluted with 2 mL of 5% ammonia in a methanol-acetonitrile mixture (30:70, *v*/*v*). The eluate was evaporated to dryness at 45 °C under a stream of nitrogen and reconstituted with 100 µL of methanol-water mixture (50/50, *v*/*v*) and analysed by LC-MS/MS.

### 3.6. Instrumentation and Chromatographic Conditions

Analysis of reconstituted DBS samples was performed on an Agilent 1290 Infinity liquid chromatographic system (Agilent Technologies, Santa Clara, CA, USA) coupled to an Agilent 6460 Triple Quad Mass Spectrometer equipped with a Jet Stream^TM^ ESI source (Agilent Technologies, Inc., Santa Clara, CA, USA), operated in the positive electrospray ionization (ESI) mode. The chromatographic separation was performed on a reversed-phase Kinetex^®^ C18 column (50 mm × 2.1 mm, 2.6 μm particles) guarded by a C18 cartridge column (4 mm × 2 mm; Phenomenex, Torrance, CA, USA). The column temperature was maintained at 50 °C, and the autosampler temperature was kept at 5 °C. The injection volume was 1.0 µL. The MS was protected by using a flow-diverter valve, which let the flow enter the MS only between 0.7 and 1.3 min; otherwise, the flow was directed to waste. The mobile phase composition was optimized to achieve the baseline resolution between LTG and LTG-N2-GLU in the shortest amount of time. The mobile phase consisted of 0.1% formic acid in MilliQ water (mobile phase A) and 100% acetonitrile (mobile phase B). The flow rate was set at 0.65 mL/min and the following gradient was employed (% of mobile phase B): 5.0, 5.0, 30, 60, 60, and 5.0 at the corresponding time points: 0, 0.25, 0.70, 1.0, 1.5, and 1.7, respectively. The total run time was 2.2 min. Instrument parameters were optimized to achieve the best sensitivity and were set as follows: drying gas temperature 275 °C, drying gas flow 5 L/min, nebulizer pressure 45 PSI (0.31 MPa), sheath gas temperature 320 °C, sheath gas flow 11 L/min, capillary entrance voltage 4000 V, nozzle voltage 1000 V. The dwell time was 25 ms. Instrument control, data acquisition, and quantification were performed by Mass Hunter Workstation software B.03.01 (Agilent Technologies, Santa Clara, CA, USA). Multiple reaction monitoring (MRM) settings for quantification were optimized using the Agilent Mass Hunter Optimizer software B.06.00 and are presented in Table 6.

### 3.7. Method Validation

The DBS method validation was performed according to the FDA guidelines for bioanalytical method validation [52]. The validated parameters were selectivity, accuracy and precision, lower limit of quantification, linearity, matrix effect, extraction efficiency, and stability. From the additional DBS-specific parameters, the effects of Hct and blood spot volume on measured analyte concentrations were evaluated.

#### 3.7.1. Selectivity

The selectivity of the developed method was assessed by analysing blank DBS samples from six healthy individuals, prepared according to the sample preparation protocol. Drug-free chromatograms were compared with those at the LLOQ to ensure that no interfering peaks were present in the biological matrix at the retention times of the analytes. 

#### 3.7.2. Linearity and Lower Limit of Quantification

Calibration standards at 11 concentration levels, from 0.1 to 20 µg/mL, were spotted on blank cards over 3 consecutive validation days. A non-weighted linear regression analysis was applied to calculate the slopes, intercepts, and determination coefficients of the calibration curves constructed as peak area ratios of analyte to IS versus analyte concentration. The lower limit of quantification (LLOQ) was defined as the lowest concentration, where the coefficient of variation (CV) and bias values did not exceed 20% and the analyte signal response was at least 5-fold higher compared to the response in the blank DBS sample.

#### 3.7.3. Accuracy and Precision

The accuracy and precision of the method were assessed by analysing the QC DBS sample replicates (*n* = 5) at low, medium, and high concentrations. The intra-day accuracy and precision were calculated on a single day, while the inter-day accuracy and precision were calculated by five determinations per concentration over three consecutive validation days. The intra- and inter-day precision and accuracy of the assay were determined as percent coefficient of variation (CV) and percent bias values, respectively.

#### 3.7.4. Matrix Effect and Recovery

The recovery was calculated by comparing the responses from (A) extracted spiked DBS samples at low and high QC concentration levels with those from (B) post-extraction blank DBS samples reconstituted with the standard solution at the same QC concentration level (A/B × 100%). 

The absence of the matrix effect was evaluated according to Matuszewski [53] as a relative matrix effect to ensure that the method’s accuracy is not compromised by matrices originating from different individuals. Five standard curves containing 6 calibrators (0.1, 0.3, 3, 7.5, 15.0, 20.0) were constructed using five different lots of blood obtained from five healthy volunteers. The slopes of the curves were determined by linear regression analysis of the peak area ratios of the analyte/IS versus nominal analyte concentrations, and the variance in slopes was calculated to evaluate whether the matrix effect influences the accuracy of our method. Slope RSD values below 4% were considered an indicator that the method is free from significant relative matrix effects.

#### 3.7.5. Haematocrit Effect and Influence of Blood Spot Volume

To evaluate the effect of Hct on the accuracy of analyte quantification, aliquots of blood with different Hct % (25, 35, 45, 55) were prepared. For each Hct, QC samples at three concentration levels were prepared and analysed in triplicate.

Drug-free blood in EDTA tubes was obtained from a healthy volunteer and centrifuged for 10 min at 3000× *g*. Afterwards, appropriate amounts of plasma were added to the concentrated human erythrocytes to obtain simulated blood with defined Hct levels. After spiking, the blood was incubated at 37 °C for 30 min with gentle agitation. Ten-microlitre spots were applied onto the DBS cards, and after drying, they were submitted for extraction and analysis. Concentrations were calculated by using regression curves constructed with DBS samples prepared with whole blood from the healthy volunteer. The obtained concentrations at different Hct levels were compared with nominal concentrations and the bias and CV (%) were calculated. Bias and CV values within ±15% were considered acceptable. 

The influence of different blood volumes (10, 20, 30, and 40 μL) on drug concentration was tested at the QC medium concentration level. Six-millilitre punches (in triplicate) were extracted from the centre of the DBS samples and extracted according to the sample preparation procedure. The measured ratio of analyte versus IS was compared to those obtained from 10 μL spots, and the CV (%) was calculated.

#### 3.7.6. Stability

The stabilities of LTG and LTG-N2-GLU in stock solution on DBS cards and extracted samples were tested at low and high QC samples. Stock solution stability (stored at 4 °C for LTG and −80 °C for LTG-N2-GLU) was estimated for the respective periods. The stability of DBS specimens was assessed by storing the cards at −20 °C, 40 °C, and room temperature for 5, 14, 21, and 28 days. Autosampler stability was evaluated by keeping the prepared QC samples in the autosampler at 5 °C for 24 h. The measured concentrations of stored samples were compared to those obtained after analysis of freshly spiked DBS samples.

## 4. Conclusions

We developed a rapid, sensitive, and specific LC-MS/MS method for the quantification of lamotrigine and its main metabolite, lamotrigine-N2-glucuronide, in DBS samples. The method covers a wide concentration range for both analytes and was successfully applied to the analysis of patients’ DBS samples. Correlation analysis showed good agreement between the plasma and DBS concentrations when the haematocrit and the blood-to-plasma partition coefficient were taken into account. Our method offers two major advantages: the use of DBS samples instead of plasma samples, which offers significant benefits in terms of sample handling and transport; and secondly, the simultaneous measurement of LTG and the metabolite-to-parent ratio, which helps to elucidate the reasons when lamotrigine concentrations are found outside its optimal therapeutic range due to enzyme inhibition or induction. This is especially important for elderly patients, who are frequently taking many concomitant medications and herbal supplements that can interfere with LTG metabolic clearance. 

## Figures and Tables

**Table 1:** Calibration parameters (mean values ± SD, *n* = 3), analytical range, and lower limit of quantification (LLOQ) precision and accuracy.

Analyte	Therapeutic Range (µg/mL)	Analytical Range (µg/mL)	LLOQ Precision (%RSD, *n* = 3)	LLOQ Accuracy (%)	Calibration Parameters		
					Intercept	Slope	r^2^
LTG	2.5–15	0.1–20	19.2	102	−0.0012 ± 0.0022	0.1683 ± 0.0092	0.9937
LTG-N2-GLU	NE	0.1–20	10.3	95.0	0.0124 ± 0.0128	1.1243 ± 0.0673	0.9927

NE—not established.

**Table 2:** Intra- and inter-day precision and accuracy for LTG and LTG-N2-GLU in DBS samples by LC-MS/MS.

Nominal Concentration (µg/mL)	Concentration Found (µg/mL)	CV (%)	Bias (%)
Intra-day (*n* = 5)			
LTG			
0.3	0.330	5.86	10.6
3.0	3.11	4.54	3.70
15	15.6	4.70	3.9
LTG-N2-GLU			
0.3	0.299	4.39	−0.20
3.0	2.86	5.79	−4.7
15	14.1	5.81	−6.0
Inter-day (*n* = 15)			
LTG			
0.3	0.331	0.23	10.4
3.0	2.95	5.91	−1.5
15	15.5	1.48	3.4
LTG-N2-GLU			
0.3	0.325	6.83	8.3
3.0	2.77	3.07	−7.8
15	14.2	0.78	−5.2

**Table 3:** Hct effect on LTG and LTG-N2-GLU concentration.

Hct (%)	Concentration (µg/mL)	LTG		LTG-N2-GLU	
		Bias (%)	CV (%)	Bias (%)	CV (%)
25	0.3	3.65	6.53	1.13	3.10
	3	5.85	6.60	1.50	9.62
	15	1.40	6.06	−0.04	3.89
35	0.3	6.57	2.34	7.41	3.37
	3	6.49	5.60	4.95	4.68
	15	4.06	0.96	0.09	1.01
45	0.3	−6.47	8.13	7.05	0.13
	3	3.36	4.33	5.17	5.23
	15	−0.43	5.97	−5.29	3.52
55	0.3	13.45	0.67	7.23	7.24
	3	2.92	4.11	8.40	2.44
	15	5.30	1.69	0.77	5.41

**Table 4:** Stability of LTG and LTG-N2-GLU in DBS samples. Results presented as a percentage of analyte determined relative to those obtained after the analysis of freshly prepared DBS samples.

Storage Conditions	LTG	LTG-N2-GLU
5 days at −20 °C	94.6–101.2	99.4–101.2
5 days at 40 °C	96.8–105.5	98.9–102.1
14 days at 25 °C	99.7–103.8	98.8–95.5
21 days at −20 °C	101.6–115.3	90.9–100.4
21 days at 25 °C	108.7–113.0	92.0–99.9
21 days at 40 °C	112.2–114.4	97.1–103.5
28 days at 25 °C	89.2–93.3	95.8–91.5

**Table 5:** LTG and its major glucuronide metabolite LTG-N2-GLU concentration in trough DBS samples and calculated metabolite-to-parent ratio (MPR) in a set of patients receiving 100 to 400 mg LTG per day. Samples were taken in steady-state, immediately before the morning dose.

Subj. Nr.	Dose [mg/day]	LTG [µg/mL]	LTG-N2-GLU [µg/mL]	MPR
1 *	100	4.61	0.37	0.080
2	200	3.14	1.41	0.449
3	250	6.42	1.63	0.254
4 *	400	3.50	2.14	0.611
5	100	1.83	0.60	0.328
6	300	6.09	2.10	0.345
7	100	1.84	0.31	0.168
8	250	3.48	1.09	0.313
9	400	9.39	3.22	0.343
Mean	233	4.48	1.43	0.320
SD	120	2.46	0.96	0.153

* outlying subjects.

**Table 6:** The MRM transitions and fragmentation parameters for optimal quantification of LTG, LTG-N2-GLU and LTG^13^C_3_ (IS).

	MRM *m/z* Transitions	Fragmentor [V]	Collision Energy [eV]
LTG	256.0 → 211.0	170	25
LTG-N2-GLU	432.1 → 256.1	90	21
LTG^13^C_3_	259.1 → 145.0	170	37

## Data Availability

The data presented in this study are available on request from the authors.
